# Telocytes in mice bone marrow: electron microscope evidence

**DOI:** 10.1111/jcmm.12337

**Published:** 2014-07-24

**Authors:** Hua Li, Hongqi Zhang, Lin Yang, Shanshan Lu, Junbo Ge

**Affiliations:** a Shanghai Institute of Cardiovascular Diseases, Zhongshan Hospital, Fudan UniversityShanghai, China; b Department of Physiology and Medicine/Cardiology, University of CaliforniaLos Angeles, CA, USA; c Department of Anatomy, Histology and Embryology, Shanghai Medical College of Fudan UniversityShanghai, China; d Key Laboratory of Medical Imaging Computing and Computer Assisted Intervention of ShanghaiShanghai, China; e Key Laboratory, Eye and ENT Hospital, Fudan UniversityShanghai, China; f Institutes of Biomedical Sciences, Fudan UniversityShanghai, China

**Keywords:** bone marrow, Telocyte, telopodes, intercellular contacts, SEM, TEM

## Abstract

Telocytes (TCs) are a novel type of interstitial cell of whom presence has been recently documented in many tissues and organs. However, whether TCs exists in bone marrow is still not reported. This study aims to find out TCs in mice bone marrow by using scanning electron microscope (SEM) and transmission electron microscope (TEM). SEM images showed that in mice bone marrow most of TCs have small spherical cell body (usually 4–6 μm diameter) with thin long telopodes (Tps; usually one to three). The longest Tp observed was about 70 μm, with an uneven calibre. Direct intercellular contacts exist between TCs. TEM shows mitochondria within dilations of Tps. Also, by TEM, we show the close spatial relations of Tps with blood vessels. In conclusion, this study provides ultrastructural evidence regarding the existence of TCs in mice bone marrow, *in situ*.

Telocytes (TCs) represent a newly discovered type of interstitial cells [1]. The defining ultrastructural feature of TCs is the presence of special thin, long and uneven calibre (moniliform) prolongations termed telopodes (Tps; for more details see www.telcytes.com). The presence of TCs has been documented in the interstitial compartments of various tissues and organs: skin [2–4], brain [5], eye [6], skeletal muscle [7,8], respiratory tract [9–12], heart [13–18], digestive system [19–22] and accessory glands of the digestive system [22–27], genital tract [28–31] and urinary tract [32–35]. Nevertheless, it was not established whether TCs reside in bone marrow. The aim of this study is to bring convincing evidence for the existence of TCs in mice bone marrow.

This study was approved by the Institutional Ethics Board of Fudan University, according to the generally accepted international standards. Ten male C57BL/6J mice aged 5 weeks (weight: 12–16 g) were killed in the research. The femurs were harvested, and then the soft tissues attached to the femurs were removed. The femurs were then cut into two halves along their longitudinal axis by microsurgical scissors in preparation for samples treatment. The specimens were handled according to scanning electron microscope (SEM) and transmission electron microscope (TEM) routine and observed under Philips XL30E SEM (Amsterdam, The Netherlands) and FEI TECAI SPIRIT TEM (Eindhoven, The Netherlands), respectively.

Scanning electron microscope images of mice bone marrow showed that TCs have round/oval small cell bodies (average diameter 4–6 μm; Figs1A, 2, 3). Also one or two (no more than three) very long Tps were observed in the studied specimens. SEM measurements showed that the longest observed Tp is of 66.5 μm (Fig.1A). Tps are of uneven calibre - consisting in an alternation of thin segments (podomers) and dilated segments (podoms; Figs1A and 2B). Figure2A shows TCs in the close vicinity of an arteriola, and also the direct contact between two TCs is seen in Figure2B. Under TEM (Fig.4), one TC with two long and thin Tps was observed in the vicinity of capillary. Mitochondria were seen in the dilated segment (podom) of one Tp.

**Figure 1:**
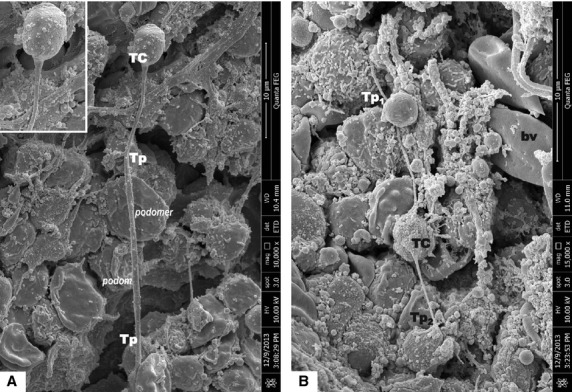
Scanning electron microscope images of mice bone marrow. (A) It shows a telocyte (TC) with one visible telopode (Tp). Cellular body of TC is round (inset), having the measured sizes of 3.55/4.5 μm. Tp have the measured length of 66.5 μm, with the distal end partially covered. The inset shows the abrupt emerging on the Tp from the cellular body of TC; bar = 30 μm. (B) TC with two long and very thin Tps: Tp1 - 14.12 μm, and Tp2 - 6.48 μm. The measured size of the cellular body of TC is 2.65/4.55 μm. The uneven calibre (alternation of podoms and podomers) of the Tps is obvious; bar = 10 μm.

**Figure 2:**
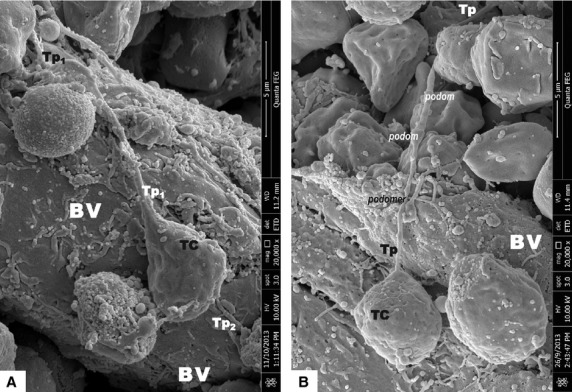
Scanning electron microscope images of mice bone marrow. (A) Telocyte (TC) and its Telopode (Tp) are located on the surface of an arteriola. TC cellular body size: 4.44/5.93 μm; Tp dimensions: length - 12.5 μm; diameter - 0.44 μm; bar = 5 μm. (B) TC with its Tp in close spatial relationship with surrounding interstitial cells. The moniliform aspect of the Tp is obvious: the alternation of dilated segments (A) with thin segments (B). TC cellular body size: 4.5 μm in diameter; Tp length - 15.12 μm (being which was partially sheltered by one cell); bar = 5 μm.

**Figure 3:**
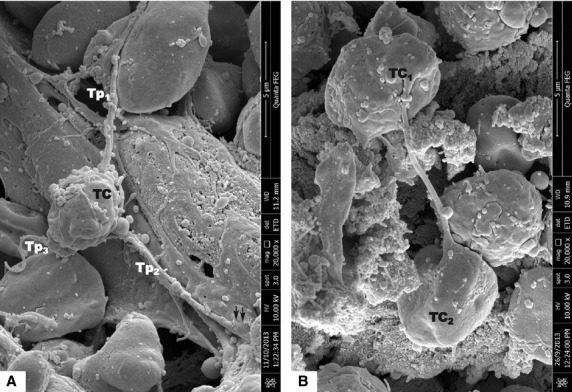
Scanning electron microscope images of mice bone marrow. (A) Telocyte (TC) with three telopodes. The cell body was oval which size was 3.53 μm in width and 4.85 μm in length. The lengths of three telopodes Tp1, Tp2 and Tp3 were 4.34, 8.12 and 8.58 μm, respectively. The terminal end of Tp2 formed contact with another cell (black dotted line circle); bar = 5 μm. (B) There was direct contact between two similar TCs in size and external appearance like dumbbell; bar = 5 μm. Telocyte-cell body, Tp-telopode.

**Figure 4:**
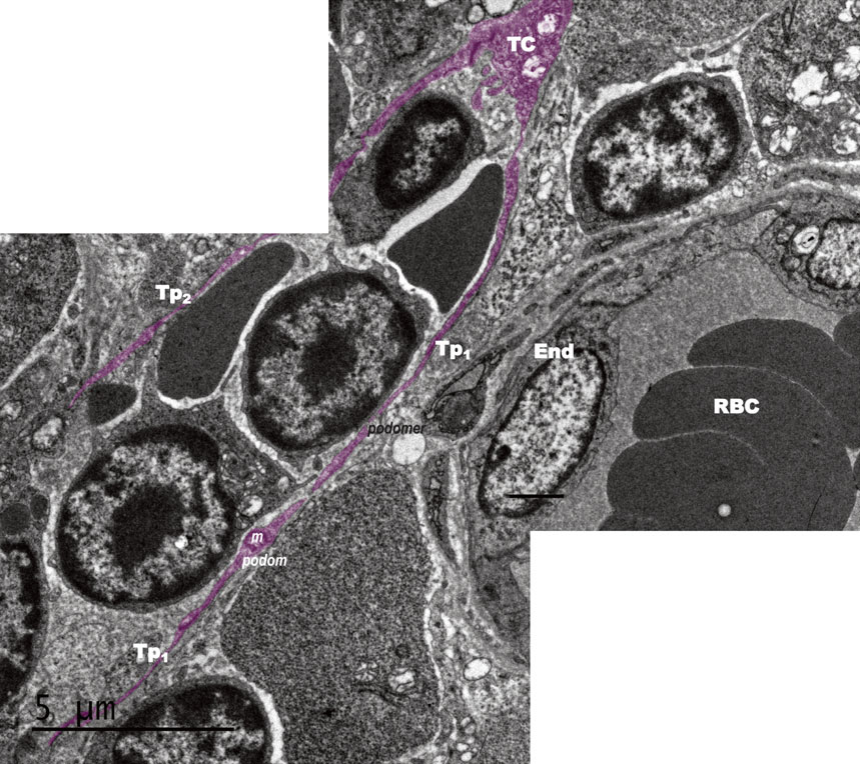
Transmission electron microscope image of mice bone marrow (combined photo). A telocyte (TC) with two long telopodes (Tps) was close to a capillary. The cell body was not totally shown. The lengths of Tp1 and Tp2 were 19.98 μm and 12.99 μm, respectively. The calibre of Tps was uneven. Mitochondria (M) was observed within the podom. Cap: capillary; L: lumen; RBC: Red blood cells; End: Endothelial cell; bar = 5 μm.

The presence of TCs has been previously documented in many organs. They were ultrastructurally described in close contacts with stem cells within interstitium of several vital organs [3,6,10,14,16,36]. However, TCs in bone marrow (stem cell abundant hematolymphoid tissue) was never reported previously. We provide here for the first time ultrastructural evidence for the presence of TCs within bone marrow. Ultrastructurally, TCs found in mice bone marrow share the similar distinctive features with TCs described by Popescu's group within other organs [1]. TCs have small cellular body with 1–3 suddenly emerging telopodes of uneven calibre. Similar to the previous reports, within bone marrow, TCs appear interconnected by homocellular junctions, forming a network. In mice bone marrow TCs are observed in close spatial relationships with small blood vessels and/or capillaries. Also, the presence of TCs in bone marrow tissue characterized by the generous presence of stem cells and progenitor cells is somehow re-capitulate the condition of TCs in stem cell niches. This aspect could offer new insights regarding the presumptive role(s) of TCs in cellular (re)generation.

## References

[1] Popescu LM, Faussone-Pellegrini MS (2010). TELOCYTES - a case of serendipity: the winding way from Interstitial Cells of Cajal (ICC), *via* Interstitial Cajal-Like Cells (ICLC) to TELOCYTES. J Cell Mol Med.

[2] Suciu L, Popescu LM, Gherghiceanu M (2010). Telocytes in human term placenta: morphology and phenotype. Cells Tissues Organs.

[3] Ceafalan L, Gherghiceanu M, Popescu LM (2012). Telocytes in human skin - are they involved in skin regeneration?. J Cell Mol Med.

[4] Rusu MC, Mirancea N, Manoiu VS (2012). Skin telocytes. Ann Anat.

[5] Manetti M, Guiducci S, Ruffo M (2013). Evidence for progressive reduction and loss of telocytes in the dermal cellular network of systemic sclerosis. J Cell Mol Med.

[6] Popescu BO, Gherghiceanu M, Kostin S (2012). Telocytes in meninges and choroid plexus. Neurosci Lett.

[7] Luesma MJ, Gherghiceanu M, Popescu LM (2013). Telocytes and stem cells in limbus and uvea of mouse eye. J Cell Mol Med.

[8] Suciu LC, Popescu BO, Kostin S (2012). Platelet-derived growth factor receptor-bpositive telocytes in skeletal muscle interstitium. J Cell Mol Med.

[9] Diaz-Flores L, Gutierrez R, Saez FJ (2013). Telocytes in neuromuscular spindles. J Cell Mol Med.

[10] Zheng Y, Bai C, Wang X (2012). Potential significance of telocytes in the pathogenesis of lung diseases. Expert Rev Respir Med.

[11] Zheng Y, Bai C, Wang X (2012). Telocyte morphologies and potential roles in diseases. J Cell Physiol.

[12] Zheng Y, Zhang M, Qian M (2013). Genetic comparison of mouse lung telocytes with mesenchymal stem cells and fibroblasts. J Cell Mol Med.

[13] Zheng Y, Cretoiu D, Yan G (2014). Comparative proteomic analysis of human lung telocytes with fibroblasts. J Cell Mol Med.

[14] Gherghiceanu M, Popescu LM (2012). Cardiac telocytes - their junctions and functional implications. Cell Tissue Res.

[15] Zhao B, Chen S, Liu J (2013). Cardiac telocytes were decreased during myocardial infarction and their therapeutic effects for ischemic heart in rat. J Cell MolMed.

[16] Bani D, Nistri S (2014). New insights into the morphogenic role of stromal cells and their relevance for regenerative medicine. lessons from the heart. J Cell Mol Med.

[17] Sheng J, Shim W, Lu J (2014). Electrophysiology of human cardiac atrial and ventricular telocytes. J Cell Mol Med.

[18] Zhao B, Liao Z, Chen S (2014). Intramyocardial transplantation of cardiac telocytes decreases myocardial infarction and improves post-infarcted cardiac function in rats. J Cell Mol Med.

[19] Yang Y, Sun W, Wu SM (2014). Telocytes in human heart valves. J Cell Mol Med.

[20] Cretoiu D, Cretoiu SM, Simionescu AA (2012). Telocytes, a distinct type of cell among the stromal cells present in the lamina propria of jejunum. Histol Histopathol.

[21] Vannucchi MG, Traini C, Manetti M (2013). Telocytes express PDGFRα in the human gastrointestinal tract. J Cell Mol Med.

[22] Milia AF, Ruffo M, Manetti M (2013). Telocytes in Crohn's disease. J Cell Mol Med.

[23] Chen X, Zheng Y, Manole CG (2013). Telocytes in human oesophagus. J Cell Mol Med.

[24] Nicolescu MI, Bucur A, Dinca O (2012). Telocytes in parotid glands. Anat Rec.

[25] Nicolescu MI, Popescu LM (2012). Telocytes in the interstitium of human exocrine pancreas: ultrastructural evidence. Pancreas.

[26] Matyja A, Gil K, Pasternak A (2013). Telocytes: new insight into the pathogenesis of gallstone disease. J Cell Mol Med.

[27] Bosco C, Díaz E, Gutiérrez R (2013). Ganglionar nervous cells and telocytes in the pancreas of Octodon degus: extra and intrapancreatic ganglionar cells and telocytes in the degus. Auton Neurosci.

[28] Xiao J, Wang F, Liu Z (2013). Telocytes in liver: electron microscopic and immunofluorescent evidence. J Cell Mol Med.

[29] Cretoiu SM, Cretoiu D, Popescu LM (2012). Human myometrium - the ultrastructural 3D network of telocytes. J Cell Mol Med.

[30] Rosenbaum ST, Svalø J, Nielsen K (2012). Immunolocalization and expression of small-conductance calcium-activated potassium channels in human myometrium. J Cell Mol Med.

[31] Hatta K, Huang ML, Weisel RD (2012). Culture of rat endometrial telocytes. J Cell Mol Med.

[32] Corradi LS, Jesus MM, Fochi RA (2013). Structural and ultrastructural evidence for telocytes in prostate stroma. J Cell Mol Med.

[33] Zheng Y, Zhu T, Lin M (2012). Telocytes in the urinary system. J Transl Med.

[34] Gevaert T, De Vos R, Van Der Aa F (2012). Identification of telocytes in the upper lamina propria of the human urinary tract. J Cell Mol Med.

[35] Qi G, Lin M, Xu M (2012). Telocytes in the human kidney cortex. J Cell Mol Med.

[36] Popescu LM, Manole E, Serboiu CS (2011). Identification of telocytes in skeletal muscle interstitium: implication for muscle regeneration. J Cell Mol Med.

